# Cervical scoliosis and torticollis: a novel skeletal anomaly in broiler chickens

**DOI:** 10.1186/s13028-019-0482-0

**Published:** 2019-10-11

**Authors:** Andrew Olkowski, Chris Wojnarowicz, Boguslaw Olkowski, Bernard Laarveld

**Affiliations:** 1 Department of Animal and Poultry Science, University of Saskatchewan, Saskatoon, SK S7N 5A8 Canada; 2 Prairie Diagnostic Services, Veterinary Pathology, University of Saskatchewan, Saskatoon, SK S7N 5A8 Canada; 3 Institute of Bioengineering and Animal Breeding, Siedlce University of Natural Sciences and Humanities, B. Prusa 14, 08-110 Siedlce, Poland

**Keywords:** Broiler chicken, Cervical scoliosis, Osteoclast, Skeletal deformity, Torticollis

## Abstract

**Background:**

Among the most prominent health problems marring the global poultry industry for several decades are skeletal abnormalities. The aim of this study was to investigate a recent emergence of a novel form of skeletal deformity affecting cervical spine in broiler chickens. This work presents the natural history of this newly emerging skeletal anomaly along with long term observations of epidemiological trends in commercial broiler flocks, and clinical and pathological features.

**Results:**

In distinction from other forms of skeletal deformities commonly reported in broiler chickens, this new form of cervical spine anomaly have been observed in newly hatched chicks and in fully developed embryos that died in the shell. On clinical and post mortem examination this condition presents characteristic features consistent with congenital cervical scoliosis and torticollis (CCST). The pathogenesis of CCST appears to be linked to pathological remodeling of the cervical vertebrae bone associated with excessive activity of osteoclasts. Long term observations indicate that the incidence of CCST showed increasing epidemiological trends over time. More recently CCST has been observed in newly hatched chicks with incidence ranging from 0.1 to > 1%, and in fully developed embryos that failed to hatch about 4 to 5%.

**Conclusions:**

The increasing trends in incidence of CCST in commercial broiler flocks are of concern from an economic perspective, and also represent a very specific and important aspect of animal welfare.

## Background

For many years the commercial broiler industry has focused on genetic selection for economically important traits such as rapid gain of muscle mass, decreased time from hatch to market, and increased feed efficiency. Undoubtedly, this strategy has resulted in the development of a chicken genotype with superior growth characteristics, but history has shown that intensive genetic selection in meat type poultry for production traits will inevitably lead to occurrence of undesirable traits [1]. In particular, the poor physical fitness predisposing chickens selected for rapid growth to skeletal disorders is a prime example of negative effects of intensive genetic selection for production traits, and represents an important aspect of the health problems which the global broiler industry has been facing continually for several decades [2–5].

In the agricultural animal production sector, genetic improvement for economically important traits is an ongoing process, and the emergence of any health problems in food producing animals is always a cause for concern. In particular, an emergence of skeletal anomalies in the meat type poultry sector needs special attention because of high selection pressure for fast growth, and rapid turnover of populations subjected to selection.

The Department of Animal and Poultry Science, University of Saskatchewan, Canada has been monitoring health problems in commercial broiler flocks for more than two decades [5–10], and this has resulted in a large data base of health-related records. A recent review of our records revealed the emergence of a new form of skeletal anomaly characterized by an abnormal posture of the neck and head. Increasing trends in the incidence of this anomaly were noted over time, and in particular over the past decade. This anomaly attracted attention not only because of its clinical novelty, but taken together with increasing incidence, this condition also has become a growing economic and animal welfare concern. Accordingly, herein we present the natural history and pathological features of this newly emerging skeletal anomaly along with long term observations of epidemiological trends in commercial broiler flocks documented by our research group.

## Methods

### General

The epidemiological trends of the emerging skeletal abnormality presented in this report are described in the context of historical records collected by our research group between 1994 and 2016. The data on the prevalence were collected from day old broiler chicks destined for various experiments at the University of Saskatchewan and from small commercial flocks in Saskatchewan, Canada. Overall, during the course of our observations we screened between 5000 and 40,000 day-old broiler chicks per year (around 350,000 in total). All birds were commercial broilers supplied by local commercial hatchery. The birds were delivered to the facility in plastic crates (100 chicks per crate). On arrival, all chicks were examined for general fitness, together with observation for overt signs of cervical spine deformities. The diagnostic criteria included abnormality of the cervical spine with the neck in a bent and/or twisted position such that the head is drawn to side, upwards or downwards. In the experimental setting, the evaluation was performed by principal investigator (PI), and in commercial situation the data was collected either through personal observation by PI or personal communications with producers or caregivers. The birds showing signs of cervical spine deformity were removed and either were euthanized or some taken to the lab for further examination.

Periodical cross-sectional studies to evaluate incidence of the cervical spine deformity in full term, unhatched chicks were conducted between 2013 and 2017 in the province of Saskatchewan, Canada (North American arena) and eastern Poland (European arena) in collaboration with local commercial hatcheries (one per location). A total of 2730 unhatched eggs were examined in 2013 and 2014 in Canada, and 2360 in Poland. The unhatched eggs were examined within 2 to 6 h after the completion of hatching process. The chicks were carefully extracted from the shell and subjected to visual evaluation for cervical spine deformity.

### Gross pathology and light microscopy

Following clinical evaluation, representative samples of affected birds and normal flock mates were euthanized using T-61 Euthanasia Solution (Intervet, ON, Canada) and subjected to post mortem evaluation. Specimens from five affected and five normal broilers were processed for detailed examination of gross and microscopic changes in cervical vertebrae and cervical spinal cord. For microscopic examination sections of the neck were fixed in phosphate buffered 10% formalin solution. Following fixation, segments of spinal cord were removed, and the bone specimens were decalcified in 20% aqueous formic acid solution. Blocks of respective tissue sections were embedded in paraffin. Longitudinal and transverse sections (5 μm in thickness) were processed for light microscopy and stained with hematoxylin and eosin (H&E) or phosphotungstic acid hematoxylin (PTAH) stains.

## Results

### General clinical observation and epidemiological trends

**Fig. 1:**
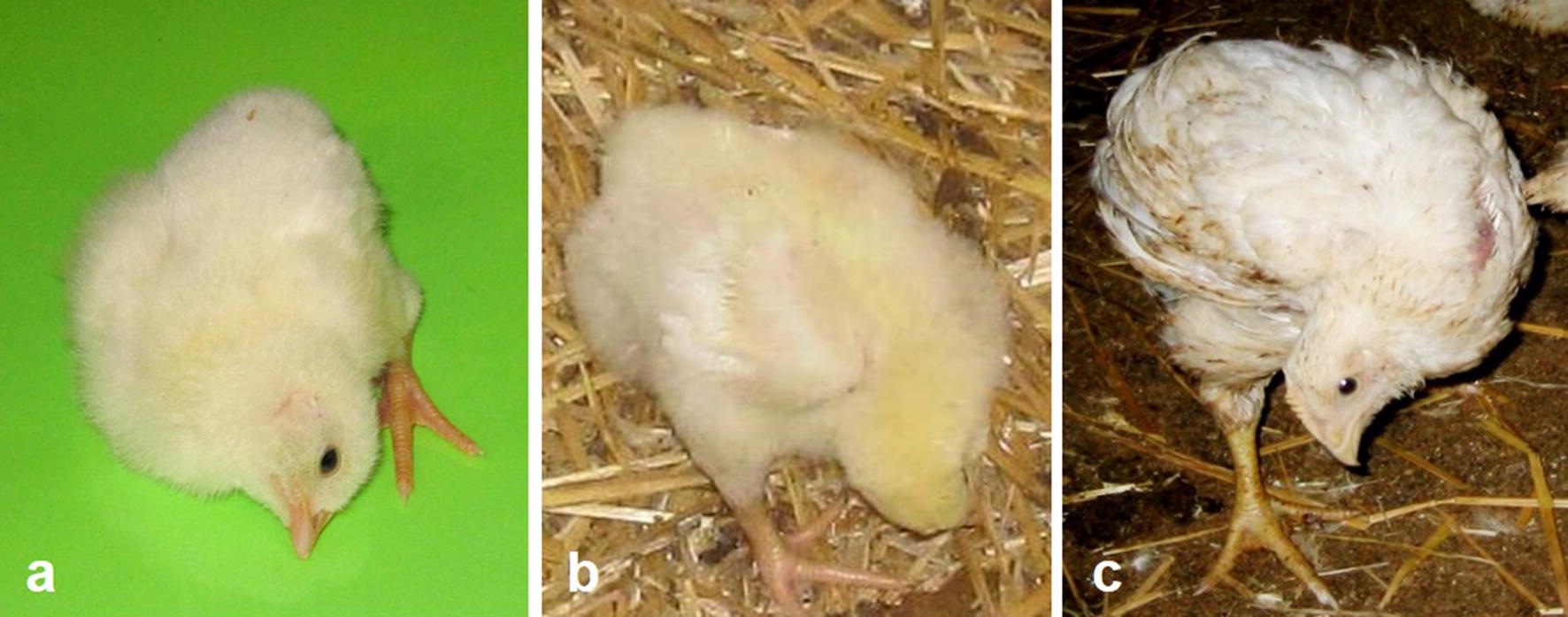
Cases of skeletal deformity of the neck observed in commercial broilers. **a** Shows severe neck deformity in a newly hatched broiler chick. **b** Shows severe neck deformity in a broiler chick found in a commercial flock on day 3 after placing. **c** Shows neck deformity in a broiler found in a market age commercial flock. Notably, despite its profound anatomical changes, this bird survived in the flock throughout the production cycle and appeared to be in good health, but was approximately 40% smaller in comparison to normal broilers from this flock

Retrospective analysis of the records collected by our research group over the last 25 years revealed emergence of a skeletal deformity affecting the cervical spine in commercial broiler flocks. Chicks affected by this condition typically show the neck in a bent and twisted position such that the head is drawn to one side, upwards or downwards. Typical examples of this skeletal deformity in newly hatched chicks and broilers at various stages of growth are shown in Fig. 1. The observed pathological features are consistent with cervical scoliosis and torticollis, and since these changes are clearly discernible in newly hatched chicks, this anomaly can be characterized as congenital cervical scoliosis and torticollis (CCST) syndrome.

**Fig. 2:**
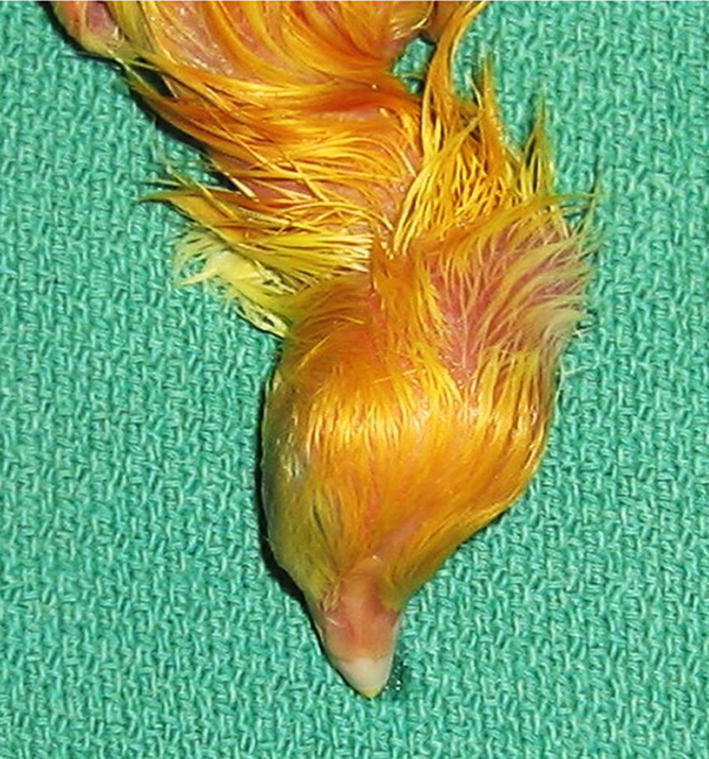
Example of typical deformity of the cervical vertebrae column observed in an otherwise well developed, full term chick that was found dead in the shell

Based on our records, the CCST syndrome in commercial broiler flocks was not observed in the past until sporadic cases were noted in the late 1990s. Since early 2000s an increase in incidence was noted over time, and in particular over the last decade an increase in the incidence of this condition has been observed in newly hatched chicks with the frequency of occurrence ranging between 0.1 and  > 1%. Cervical spine scoliosis and torticollis was also observed in many full term, unhatched, dead in the shell chicks (example shown in Fig. 2). Further examination of large populations of unhatched eggs (n = 5090) revealed that approximately 4 to 5% of well-developed, otherwise normal chicks that died in the shell showed features characteristic of CCST (Table 1).

**Table 1:** Incidence of congenital deformity of cervical spine in apparently normal, dead in the shell, full term chick embryos observed in commercial hatcheries

Item	Unhatched eggs examined	Dead in shell embryos showing signs of cervical scoliosis and torticollis
North American study^a^	2730	132 (4.8)
European study^b^	2360	93 (4.0)

Numbers in parentheses indicate % examined

^a^Study conducted in the province of Saskatchewan, Canada

^b^Study conducted around Siedlce district, Eastern Poland

### Gross pathology and histopathology

**Fig. 3:**
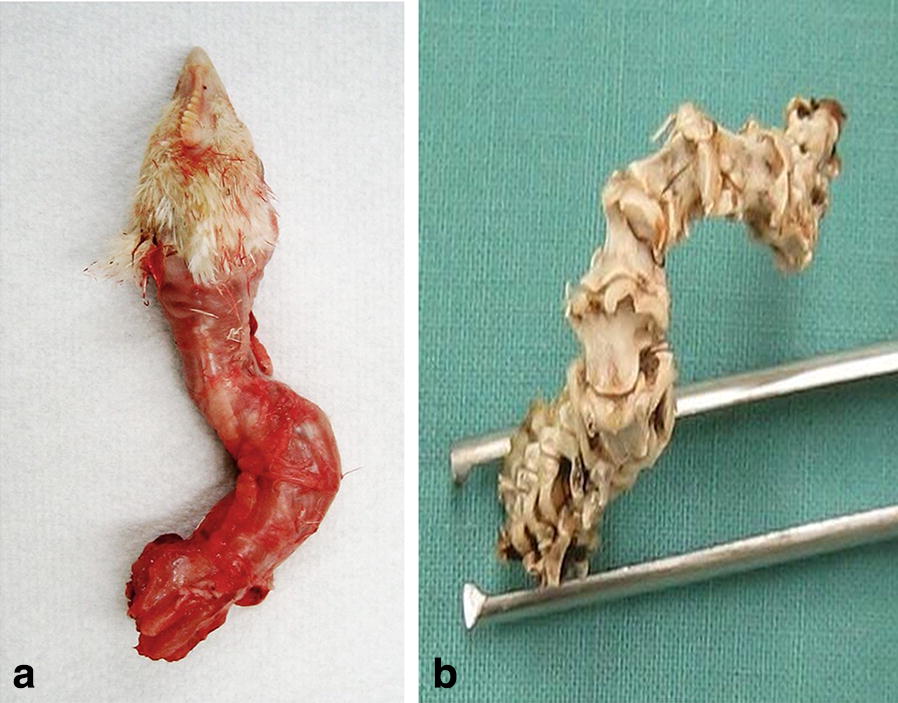
Gross pathological changes of the neck from a commercial broiler showing clinical signs of severe cervical spine deformity. Noteworthy are skeletal changes in the specimen with intact muscular anatomy shown in topical view (**a**) and cervical spine following removal of soft tissue (**b**). In order to facilitate removal of soft tissue, the freshly extracted neck was boiled in water, and the muscle tissue was carefully separated from the bone while paying attention to avoid damage to intervertebral ligaments. This technique allowed us to extract skeletal structures of the neck with totally preserved anatomical integrity of the cervical vertebrae column

All cases of CCST showed similar general gross pathological features, only differing in the neck and head deformity orientation (head drown towards left or right side, bent or twisted upwards or downward), and magnitude of the changes. Gross pathological changes of the neck of commercial broilers showing clinical signs of severe cervical spine deformity are shown in Fig. 3. Noteworthy are skeletal changes in the specimen with intact muscular anatomy shown in topical view (Fig. 3a) and cervical spine following removal of soft tissue (Fig. 3b) characteristic of scoliosis with helical twist characteristic of torticollis.

**Fig. 4:**
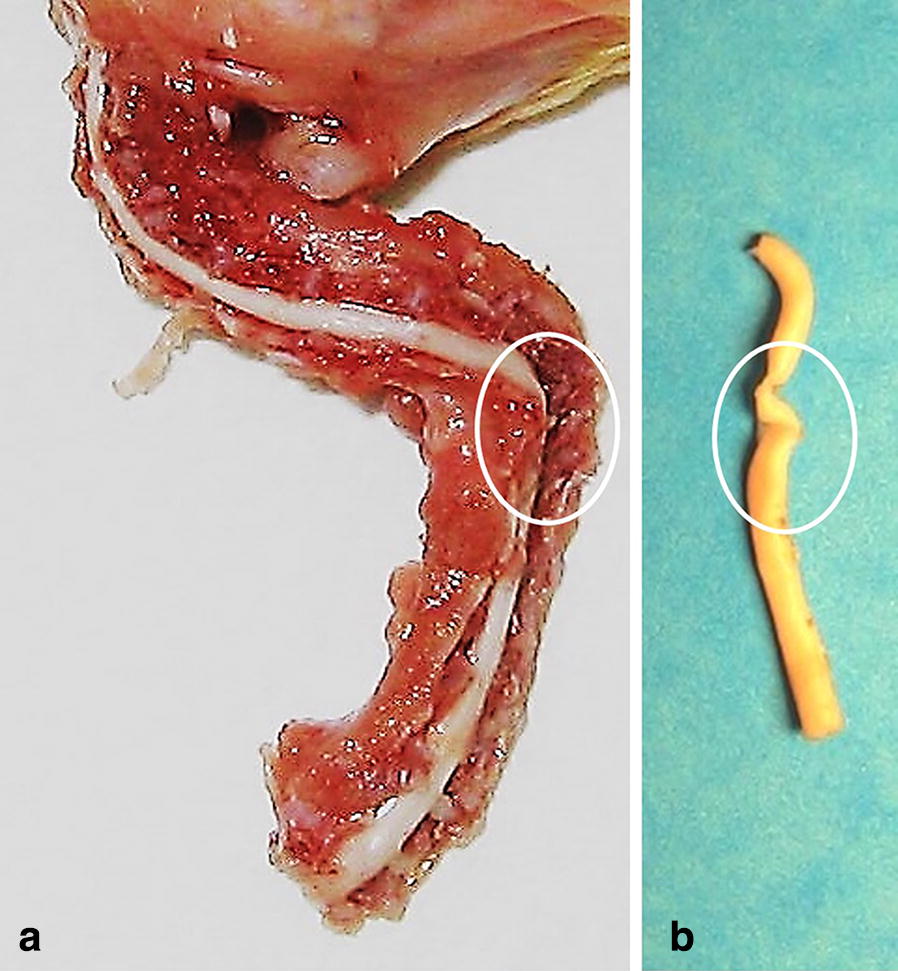
Gross pathological features of the neck from a commercial broiler showing severe clinical signs of cervical spine deformity. **a** Shows topical view of the neck with preserved muscular anatomy with cross section showing exposed spinal cord. Noteworthy is a helical twist of the vertebral column (**a**, circle). The specimen shown in **a** was next preserved in formalin, and following fixation the spinal cord was extracted (**b**). Noteworthy are changes in spinal cord anatomy caused by cervical vertebrae deformity (**b**, circle)

**Fig. 5:**
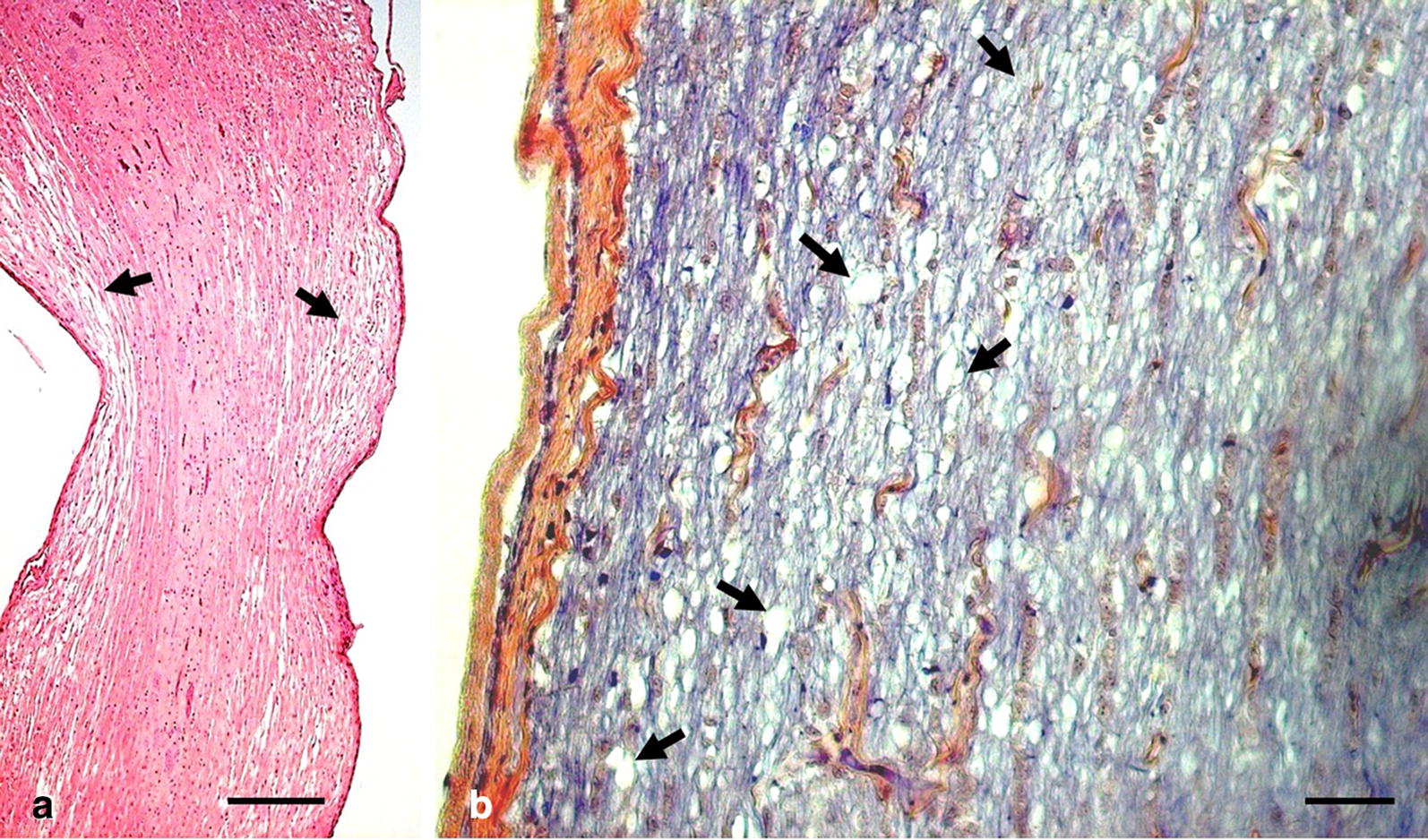
Histo-pathological features of spinal cord tissue from a commercial broiler showing clinical signs of severe cervical spine deformity. Micrographs show the longitudinal topography of the spinal cord tissue taken from the section compressed by deformed cervical vertebral column (Fig. 4b). The section at sub-gross level (**a**, stained with H&E) indicates loss of myelin in the areas directly affected by deformation (arrows), which is further confirmed at high magnification (**b**, stained with PTAH). Noteworthy are multifocal and locally extensive areas of myelin loss (arrows) and localized rarefication of glial fibers. Bars: **a**: 500 µm; **b**: 40 µm

In more severe cases, the deformity of the cervical spine (Fig. 4a) also affected the morphology of the spinal cord (Fig. 4b). Microscopic examination of sections from the affected area of the spinal cord shown in Fig. 4b revealed multifocal and locally extensive areas of myelin loss and localized rarefication of glial fibers (Fig. 5). Interestingly however, there were no apparent changes in motor neurons in the affected areas of the spinal cord. There were no apparent gross or microscopic changes in the muscles of the cervical spine.

**Fig. 6:**
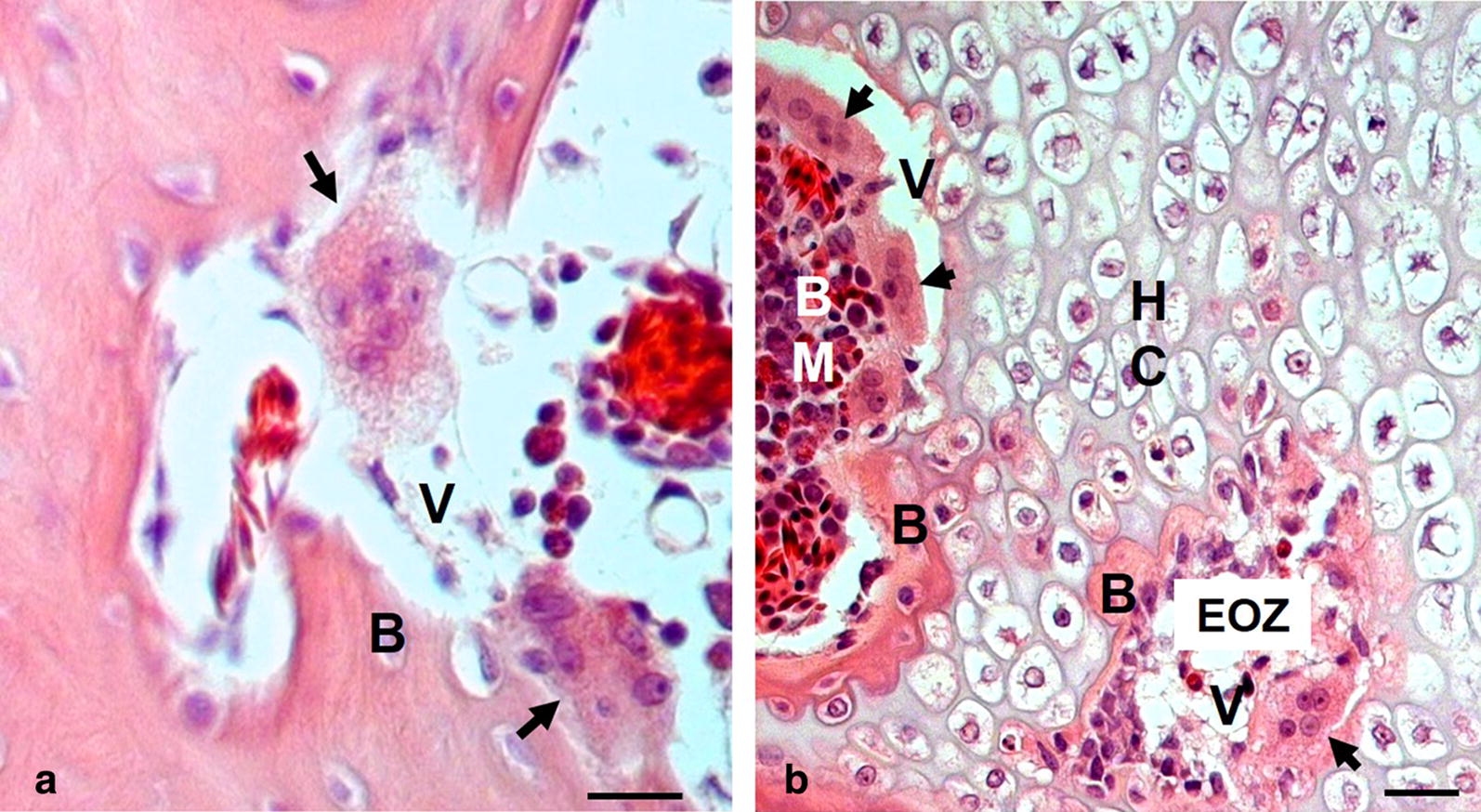
Microscopic images of cervical vertebrae sections from a broiler chicken with clinical signs of cervical spine deformity. **a** Erosions in newly formed bone structure associated with aggressive action of pathologically altered osteoclasts (*arrows*). Noteworthy are extensive amorphous vacuoles (V) in the areas previously occupied by bone structure (B). **b** cervical vertebrae hypertrophic cartilage (HC) with osteolysis of newly formed bone structure (B) at the interface of calcification zones of cartilage and bone marrow cavity (BM), as well as newly formed bone in the areas of endochondral ossification zone (EOZ) associated with aggressive action of pathologically altered osteoclasts, which are considerably enlarged and contain numerous nuclei (arrows). Noteworthy are clusters of osteoclasts lining the interface of bone marrow cavity and calcification zone of cartilage and penetrating the endochondral bone (arrows). It is apparent that the action of osteoclasts prevented bone ossification leaving amorphous vacuoles (V) in the areas that should be filled by newly formed bone structure (B). Bars: 20 µm

Microscopic evaluation of cervical vertebrae bone typically showed increased numbers of pathologically altered osteoclasts (Fig. 6). These features were not seen in specimens from normal chickens. Therefore, the increased activity of osteoclasts in the deformed areas of cervical vertebrae appears to be associated with the excessive resorption of bone (Fig. 6a). Interestingly, bone resorption appears to commence as soon as the new bone is formed, where it is clearly discernible in the areas of primary ossification centers and endochondral bone formation (Fig. 6b). Noteworthy are the activities of osteoclasts in the cartilage zone of proliferation, hypertrophic zone, and zone of calcification, as well as in areas where new bone was already formed.

## Discussion

Over the entire history of intensive genetic selection for economically important targets, the broiler industry has witnessed a plethora of health problems. Undoubtedly, among the most prominent health problems marring the global poultry industry for several decades are skeletal abnormalities [2, 3, 5]. The deformity affecting the cervical spine described in the present report exemplifies a new category of skeletal disorders in broiler chickens.

Idiopathic deformities of the spine in chickens described as scoliosis were reported previously [11], but in these cases the deformity involved the thoracic vertebrae. However, idiopathic cases of torticollis were not reported previously, and reported cases of torticollis were always associated with some causative factor including bacterial infections [12, 13], yeast infection [14], parasitic infections [15], viral diseases including Marek’s disease [16], Newcastle disease [17], avian paramyxovirus [18], enteric reovirus strains [19], and avian influenza virus [20, 21]. Torticollis was also observed in broilers fed soybean based diets [22] and lupine based diets [23]. Furthermore, the cases of torticollis reported in avian medicine literature have been described primarily in older birds. Notably, in contrast to previous reports, torticollis in the present study was observed in newly hatched chicks, and had no apparent association with any known foregoing etiology.

To the best of our knowledge, idiopathic cervical scoliosis in combination with torticollis has not been described previously in chickens. At present, the pathogenesis of this novel form of scoliosis/torticollis is not clear. However, it is noteworthy that the studies on etiology of various forms of thoracic scoliosis in chickens have shown that its etiology includes a significant genetic component [24]. Given that the deformity of the cervical spine observed in our studies has attributes of a congenital condition, the genetic predisposition to this anomaly warrants further investigation.

The potential pathophysiological factor that merits consideration is the pineal gland inadequacy, as several studies have shown that in broiler chickens scoliosis can be induced experimentally by removal of the pineal gland [25–27]. In the context of increased activity of osteoclasts observed in the vertebral bone of the affected chicks, of particular interest is the work of Yoshihara et al. [27] where the authors noted that vertebral deformity induced by pinealectomy was associated with increased activity of osteoclasts, and the authors attributed this activity to the development of spinal column deformity through the changes in bone modeling. Since the pineal gland is the primary source of melatonin, it is possible the melatonin insufficiency may be involved in the etiology of cervical bone changes during embryonic development. Interestingly, it has been shown that melatonin inhibits osteoclast formation and activation [28], whereas lack of melatonin promotes osteoclast proliferation [27]. Notably, in the present study, in comparison to normal chickens, osteoclasts seen in the cervical vertebral bone of the affected broilers were increased in numbers. They are also enlarged and contain a large number of nuclei, which are attributes of osteoclasts pathology (for review see [29, 30]). These features were absent in specimens obtained from normal chickens of similar age. Furthermore, unlike in normal chickens, cervical vertebrae from chickens affected with CCST showed osteoclast activity in the cartilage zone of proliferation, hypertrophic zone, and zone of calcification, as well as in areas where new bone was formed. Taken together, these findings suggest that the cervical vertebrae bone metabolism in the affected broilers involves both impaired bone formation and increased resorption activity. Such changes, which are particularly prominent in the zones where new bone is formed, indicate pathological bone development and remodeling, which is likely associated with activation of osteoclasts. Of note, our observations on increased activity of osteoclasts in cervical vertebral bone from affected broilers bear a similarity with the findings from studies in broiler chickens where scoliosis was induced experimentally by pinealectomy [27, 31]. Hence, the possibility that cervical spine deformity observed in our study may be linked to genetic defect of pineal gland metabolism warrants further investigation.

## Conclusion

Congenital cervical scoliosis and torticollis syndrome represents a new form of skeletal disorder in broiler chickens. The increasing trends in the incidence noted in commercial broiler flocks indicate that this new skeletal anomaly should be viewed as an emerging concern. Further work should be directed towards the investigation of possible genetic and/or pathophysiological factors involved in the pathogenesis.

## Data Availability

The datasets are available from the corresponding author on reasonable request.
